# Laboratory Study of Dynamic Durability and Material Properties of Bio-Cemented Sand for Green Road Base Applications

**DOI:** 10.3390/ma18174178

**Published:** 2025-09-05

**Authors:** Fuerhaiti Ainiwaer, Tianqi Hou, Rongsong Huang, Jie Li, Lin Fan, Weixing Bao

**Affiliations:** 1 School of Highway, Chang’an University, Xi’an 710064, China; purhat@chd.edu.cn (F.A.); 2024021001@chd.edu.cn (T.H.); 15115977975@163.com (R.H.); kaishifendou2024@163.com (L.F.); 2 Xinjiang Naba Expressway Development Co., Ltd., Korla 841312, China; T2668237282@163.com

**Keywords:** microbial induced carbonate precipitation (MICP), bio-cemented sand, dynamic durability, road performance, road base material

## Abstract

Microbial Induced Carbonate Precipitation (MICP) is regarded as a promising eco-friendly alternative to traditional Portland cement for soil stabilization. However, the feasibility of applying bio-cemented soil as a road base material remains inadequately studied, particularly in terms of the relationships between MICP treatment parameters—such as solution content, curing age, and the ratio of bacterial solution (BS) to cementation solution (CS) —and key mechanical and durability properties under realistic road conditions. In this study, an optimal curing condition for bio-cemented sand was first determined through unconfined compression strength (UCS) tests and calcium carbonate content (CCC) determination. Subsequently, dynamic triaxial tests were conducted to evaluate its resistance to cyclic loading. Further road performance tests, including splitting tensile strength, freeze-thaw resistance, temperature shrinkage, and arch expansion assessments, were carried out to comprehensively evaluate the material’s applicability. Scanning electron microscopy (SEM) was employed to elucidate the microstructural mechanisms underlying strength development. The results show that the strength (4.28 MPa) of bio-cemented sand cured under optimal conditions (12% bio-cured solution content, a BS-to-CS ratio of 1:4 and 7-d curing age) satisfies the criteria for road base applications. MICP treatment significantly improved the dynamic properties of aeolian sand (AS), reducing the cumulative plastic axial strain (*ε_p_*) by nearly 11–46% and increasing the dynamic elastic modulus (*E_d_*) by approximately 7–31% compared to untreated sand. The material also demonstrates satisfactory performance across all four road performance metrics. Microstructural analysis reveals enhanced interparticle bonding due to calcium carbonate precipitation, with samples prepared near the optimum moisture content exhibiting superior integrity and strength. Overall, bio-cemented sand demonstrates excellent potential as a sustainable road base material. These findings provide a theoretical foundation for practical applications of similar bio-cemented soils in road engineering.

## 1. Introduction

Global highway construction in desert regions is currently at a development stage prioritizing both technological breakthroughs and ecological coordination. However, desert areas exhibit extreme climatic conditions, complex geological formations, and severe scarcity of high-quality road construction materials. Aeolian sand, as a locally abundant natural resource, is characterized by loose particles, lack of cohesion, and low bearing capacity, resulting in suboptimal engineering properties that necessitate stabilization treatment before use as a road base material. Traditional stabilization relies on inorganic binders represented by cement [1,2]. Nevertheless, cement production emits substantial CO_2_ and other greenhouse gases [3,4], and excessive reliance on cement in engineering will cause significant negative environmental impacts [5,6] contradicting the strategic objectives of “carbon peak and carbon neutrality”. While polymer-based organic stabilizers can achieve effective stabilization, they often entail risks of chemical pollution [7,8,9].

Currently, growing global commitment to sustainable development has drawn increasing attention to sustainability issues in geotechnical engineering. Numerous researchers have begun exploring green improvement methods for aeolian sand. Geopolymers, which utilize alkaline activators (e.g., NaOH, water glass) to activate cementitious materials (e.g., slag, fly ash), offer notable advantages in enhancing mechanical properties and promoting resource efficiency. However, in saline-alkaline environments, alkaline activators may react with external ions, further reducing soil durability [10]. Fiber-based materials can effectively improve the split tensile strength and shear resistance of aeolian sand, though their effect on compressive strength remains limited [11,12]. Biopolymers, with sodium alginate as a representative example, have been shown to enhance mechanical strength and water retention properties of aeolian sand, but their long-term durability has not yet been thoroughly validated [13,14]. Therefore, developing a green and efficient sand stabilization material or technology represents an urgent challenge in contemporary desert road construction.

Consequently, developing green and efficient sand stabilization materials or technologies constitutes a critical challenge requiring urgent resolution in contemporary desert road construction. In recent years, microbial-induced carbonate precipitation (MICP) technology has been extensively researched and applied in geotechnical engineering, including soil stabilization [15,16,17], treatment of heavy metal contaminated soil [18,19,20], and concrete crack repair [21,22,23]. Compared with conventional soil improvement methods, MICP offers advantages including environmental friendliness, high efficiency, operational simplicity, and broad applicability. Primary MICP application methods comprise three categories: surface spraying [24,25], premixing [26,27], and injection [15,16]. Among these, the injection method has received the most extensive research attention. Despite continuous refinement and in-depth study, this method still faces the challenge of uneven soil reinforcement [28,29], a critical limitation for roadbed engineering that readily induces uneven settlement and other subgrade diseases [30]. Additionally, the injection method typically requires multiple rounds of bacterial solution (BS) and cementation solution (CS) injections, resulting in substantial material consumption. Surface spraying is primarily employed for slope erosion control [31,32] and dust management [33,34]. However, this method offers limited treatment depth and yields relatively low post-treatment soil strength. In contrast, the premixing method involves thorough mixing of BS and CS with soil either sequentially or simultaneously, reducing processing time, enhancing material utilization efficiency, and effectively preventing uneven soil reinforcement. Studies [35,36] have demonstrated that the premixing method can achieve effective reinforcement outcomes. Therefore, from economic and efficiency perspectives, applying the premixing method for MICP stabilization of aeolian sand offers significant advantages.

So far, laboratory and field investigations have demonstrated the effectiveness of MICP in stabilizing aeolian sand. Studies [37,38] have demonstrated that MICP treatment facilitates the formation of cementitious calcium carbonate between loose sand particles and on their surfaces, resulting in increased unconfined compressive strength (UCS), decreased permeability coefficient, and improved wind erosion resistance in aeolian sand. Duo et al. [39] treated AS with CS at varying concentrations, revealing that UCS and CCC of the bio-cemented sand increased with higher CS concentrations. Utilizing bio-cemented sand as a base course material not only addresses the scarcity of high-quality road construction materials in desert regions but also enables the efficient, in situ utilization of aeolian sand resources. This approach reduces the demand for high-carbon-emission materials and promotes eco-friendly and sustainable practices in geotechnical engineering.

However, current research on the mechanical properties of bio-cemented sand primarily focuses on static characteristics, such as UCS [28,40] and shear strength [15,41]. These studies confirm that MICP effectively enhances the mechanical strength of AS. Research on the road performance of bio-cemented sand base layers remains scarce. Furthermore, when employed as road base material, bio-cemented sand will undergo repeated traffic loading during service [42,43], potentially leading to long-term performance degradation. Therefore, to minimize construction risks for desert highways and provide a reliable basis for promoting this novel eco-technology, it is imperative to systematically evaluate key indicators of bio-cemented sand, including mechanical strength, durability, and road performance characteristics. Accordingly, this study comprehensively investigates the feasibility of bio-cemented sand as a road base material through UCS tests, dynamic triaxial tests, and a series of road performance tests. It is expected that this study will not only advance practical applications of MICP technology in desert highway engineering but also contribute to the development of sustainable green geotechnical engineering practices.

## 2. Materials and Methods

### 2.1. Experimental Materials

#### 2.1.1. Bacterial Solution (BS) and Cementation Solution (CS)

The bacterium strain used in this study was *Sporosarcina pasteurii* (No. ATCC 11859). This strain exhibits high urease activity and operates through a straightforward urea hydrolysis mechanism that is facile to implement [44]. The BS was obtained by activating the culture in a liquid medium cultivation, which contained 15 g/L casein peptone, 5 g/L soybean peptone, 5 g/L sodium chloride, and 20 g/L urea. The pH of the medium was adjusted to 7.3 using a 1 mol/L NaOH solution, followed by sterilization in an autoclave at 121 °C for 20 min. The strains were inoculated into the liquid medium according to a volume fraction of 1% in an ultra-clean workbench and incubated in a constant-temperature shaker at 30 °C and 180 rpm for approximately 36 h. The initial absorbance (OD600 = 1.8 ± 0.2) of the bacterial suspension was measured at a wavelength of 600 nm by a spectrophotometer, and bacterial activity was determined as 4.25 mM urea hydrolyzed per minute using a conductivity meter according to the methodology established in [45]. The CS was prepared by mixing equimolar concentrations (2 mol/L) of calcium chloride and urea solutions, the former providing calcium ions for nucleation sites, while the latter served as a nitrogen source for microbial metabolism.

#### 2.1.2. Aeolian Sand (AS)

**Figure 1:**
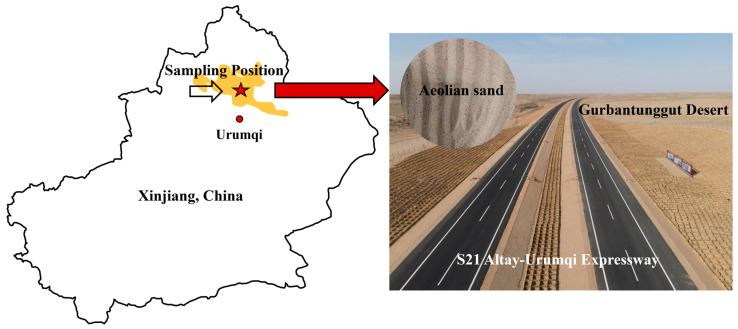
Sampling position of aeolian sand in Xinjiang, China.

**Figure 2:**
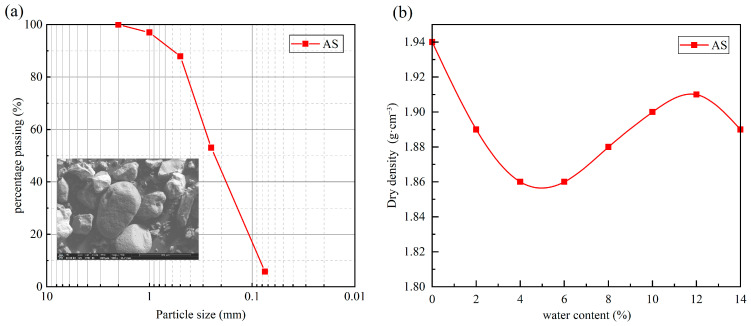
(**a**) Particle size distribution of aeolian sand; (**b**) compaction curve of aeolian sand.

The AS used in this study was taken from a soil extraction site near the S21 Aktay-Urumqi expressway in Xinjiang, China, which is located in the hinterland of the Gurbantunggut Desert, and the sampling location is shown in Figure 1. Where characterized by harsh climatic conditions featuring perennial aridity and high evaporation rates. According to the standard of geotechnical test methods (GB/T 50123-2019) [46], the particle size distribution curve and the compaction curve of AS are shown in Figure 2. The basic physical properties are demonstrated in Table 1. Based on the standard for engineering classification of soil (GB/T 50145-2007) [47], this sand is classified as silty sand (SF).

### 2.2. Sample Preparation

**Figure 3:**
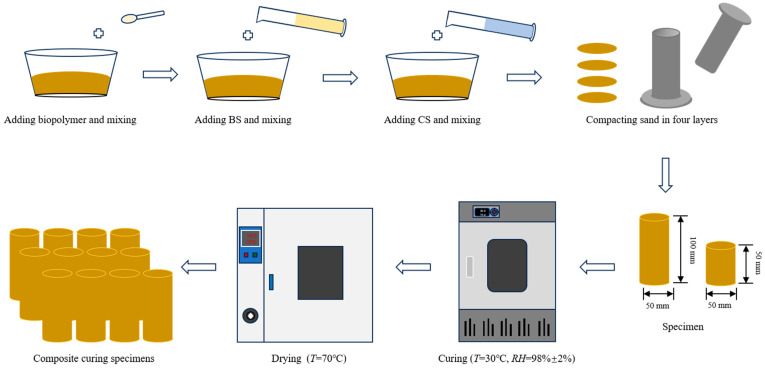
Schematic diagram of bio-cementation sand samples preparation procedure.

First, the sand was sieved through a 2 mm mesh and oven-dried at 105 °C for 24 h before cooling to room temperature. The weighed sand was transferred to a plastic basin, where predetermined volumes of BS and CS were sequentially mixed thoroughly with the sand to ensure uniform distribution. Since all stabilizers used in the experiment were in liquid form, the bio-cementation solution was utilized to replace the pure water typically required for sample preparation. The volume added corresponded to the target water con-tent of the aeolian sand samples. Uncemented sand columns served as blank controls, with specific mix design proportions detailed in Table 2. Stainless steel tubes with a 2 mm wall thickness were used as molds. The inner walls were coated with petroleum jelly-bonded oilcloth to facilitate demolding. Sand columns with dimensions of Φ50 mm × 100 mm and Φ50 mm × 50 mm were fabricated using the heavy compaction method [12]. To simulate field compaction quality, the compaction degree was controlled at 95% relative to the maximum dry density of the aeolian sand [48]. The former was designated for UCS and dynamic triaxial tests, while the latter was used for splitting tensile strength tests. Notably, chemical reactions between microorganisms and calcium sources commenced immediately during the sample preparation. To ensure uniform reinforcement levels within each batch, the preparation time was strictly limited to 1 h. After sample preparation, the sand columns were cured in a standard environmental chamber at 30 °C with 98 ± 2% relative humidity. To simulate the extreme arid conditions of desert environments, upon reaching designated curing ages, samples were oven-dried to constant weight to terminate the MICP reaction and demolded for subsequent testing. The complete operational workflow is illustrated in Figure 3.

### 2.3. Unconfined Compressive Strength (UCS) Test

The microcomputer-controlled electronic universal testing machine (WDW-100D) was used to carry out a UCS test on bio-cemented sand columns under different curing conditions. Both ends of the sand columns were polished and smoothed before the tests. The test loading speed was set at a constant rate of 1 mm/min (GB/T 50123-2019) and the maximum axial stress recorded at the point of failure was considered as the UCS for each sample. There were three parallel samples for each type of sand column, and the average value of them was taken as the final result.

### 2.4. Calcium Carbonate Content (CCC) Determination

The *CCC* value of bio-cemented sand was determined by the acid washing method [38]. First, the bio-cemented sand column was dried in an oven at 105 °C, then it was crushed and immersed in excess 1 mol/L dilute hydrochloric acid solution until no bubbles were produced. The sand particles were filtered and repeatedly washed with deionized water several times, then dried again and weighed. The mass of precipitated CaCO_3_ was calculated from the mass difference before and after acid treatment. All the tests were repeated three times to ensure reliability. The calculation formula is shown in Equation (1):

(1) $$CCC=\frac{m_{0}-m_{1}}{m_{1}}\times 100\%$$

where *m*_0_ and *m*_1_ are the dry mass of the bio-cemented sand before and after acid washing, respectively, g.

### 2.5. Dynamic Triaxial Test

**Figure 4:**
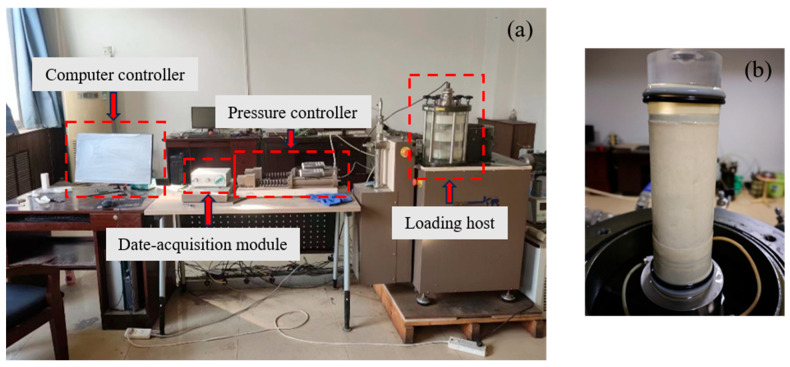
Dynamic triaxial test: (**a**) dynamic triaxial test device; (**b**) sample installation.

**Figure 5:**
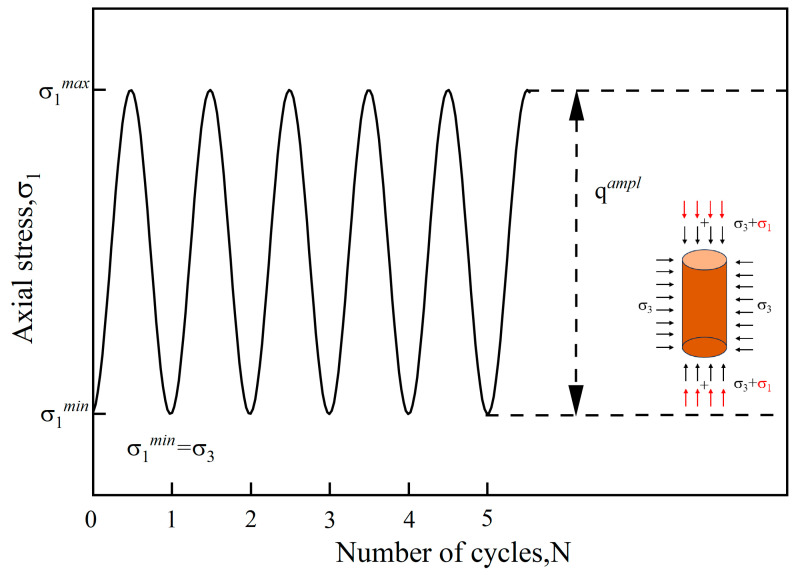
Schematic diagram of cyclic loads wave in this study.

Based on a comprehensive consideration of the bio-cemented sand’s material characteristics and the requirements for simulating actual field conditions, this study employed the GDS dynamic triaxial system (DYNTTS) to conduct dynamic triaxial tests under unconsolidated undrained (UU) conditions. The test device and sample installation can be seen in Figure 4. In order to simulate the actual working conditions of traffic loading, the half-sine wave continuous loading mode was selected in the test, and the loading waveform is shown in Figure 5. Combining the actual working conditions with the existing research [49,50], the loading frequency was 1 Hz, the number of cyclic loads was 10,000, and the confining pressures (*σ*_3_) were 50 kPa, 100 kPa, and 150 kPa, respectively, in this study. Axial stress (*σ*_1_) is the stress applied along the vertical direction of the specimen. The cyclic stress ratio (*CSR*) was defined as the ratio of cyclic bias stress (*q^ampl^*) and effective confining pressure, as shown in Equation (2):

(2) $$CSR=\frac{q^{ampl}}{\sigma_{3}}$$

where $q^{ampl}=\sigma_{1}^{\max}-\sigma_{1}^{\min}$.

When the number of cyclic loads reached 10,000 or the *ε_p_* of the sample reached 5%, the test was completed, and the specific test scheme is shown in Table 3.

### 2.6. Road Performance Tests

#### 2.6.1. Splitting Tensile Strength Test

The splitting tensile strength of bio-cemented sand columns under optimal curing conditions was determined in accordance with the Chinese standard “Test Methods of Materials Stabilized with Inorganic Binders for Highway Engineering” (JTG 3441-2024) [51]. Sample preparation followed identical procedures to those employed in the UCS test, with the exception of sample dimensions. The loading rate was controlled at 1 mm/min. Experimental results represent the effective average value obtained from six parallel samples. The strength values were calculated using the standard formulation provided in Equation (3):

(3) $$\sigma =\frac{2P}{\pi dL}$$

where *σ* is the value of the splitting tensile strength, MPa; *P* is the maximum destructive load of the sample, MPa; *d* is the diameter of the sample, mm; *L* is the total length of the sample, mm.

#### 2.6.2. Freeze-Thaw Test

**Figure 6:**
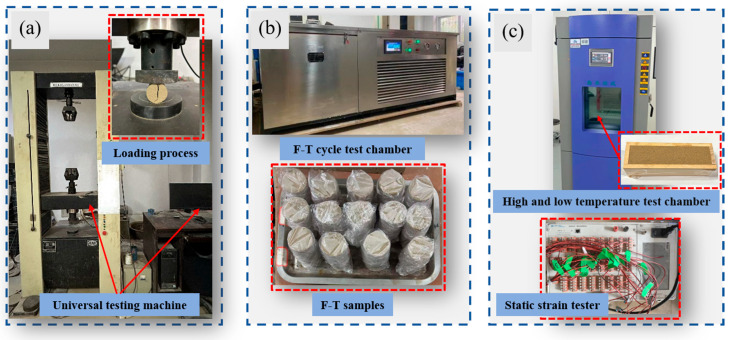
Road performance test instruments and test samples: (**a**) Splitting tensile strength test; (**b**) F-T test; (**c**) Temperature shrinkage test and arch expansion test.

Following sample curing, to prevent moisture evaporation and mass loss, samples were sealed with plastic wrap prior to freeze–thaw cycling. Freezing was conducted via three-dimensional freezing, with parameters established in accordance with Chinese standard JTG/3441-2024 alongside local climatic data. The freezing temperature was set at −20 °C for 12 h, followed by thawing at 20 °C for 12 h in a constant-temperature chamber, which is a freeze–thaw cycle. The experimental freeze–thaw chamber is illustrated in Figure 6b. After completing predetermined freeze–thaw cycles, samples were dried at 70 °C for 24 h before undergoing a UCS test.

#### 2.6.3. Temperature Shrinkage Test and Arch Expansion Test

**Figure 7:**
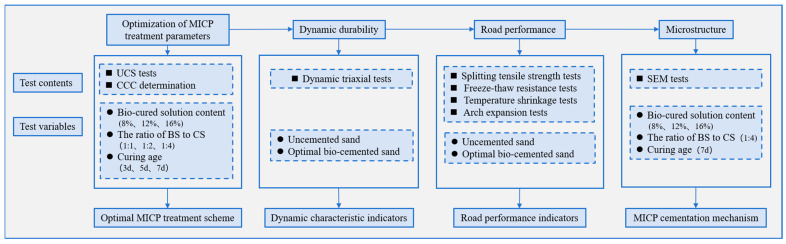
Experimental study plan flowchart.

According to the Chinese standard “Test Methods of Materials Stabilized with Inorganic Binders for Highway Engineering” (JTG/3441-2024), beam-shaped samples with the size of 50 mm × 50 mm × 200 mm were made. The environmental test equipment used for this test is the Qinzhuo programmable high and low temperature test chamber, as shown in Figure 6c. The temperature range of the test was set from −20 °C to 60 °C with a gradient of 10 °C, and each temperature interval was maintained for 3 h. The sample deformation was measured by resistive strain gages (resistance value of 120 Ω, scale distance of 80 mm), and the strain acquisition equipment was a Donghua DH3818Y static strain tester. Before the test, both sides of the sample strain gages were polished and smoothed, and then the center of the sample was calibrated, and the strain gages were pasted along the long axis; the strain gages were connected to the collector through the wires, the sample was placed in the test chamber, and the test was started after the temperature change procedure of the chamber was set up in accordance with the test program. The calculation method of the temperature shrinkage coefficient and expansion coefficient is shown in Equation (4).

(4) $$\alpha_{i}=\frac{\varepsilon_{i}}{t_{i}-t_{i-1}}$$

where *ε_i_* is the average contraction or expansion strain at the *i*-th temperature, %; *t_i_* the *i*-th temperature interval set by the temperature control program, °C; *α_i_* is the temperature contraction coefficient or expansion coefficient, which refers to the line contraction or expansion coefficient of the material under the unit temperature change. The experimental plan of this study is shown in Figure 7.

## 3. Results and Discussion

### 3.1. Unconfined Compression Strength (UCS)

**Figure 8:**
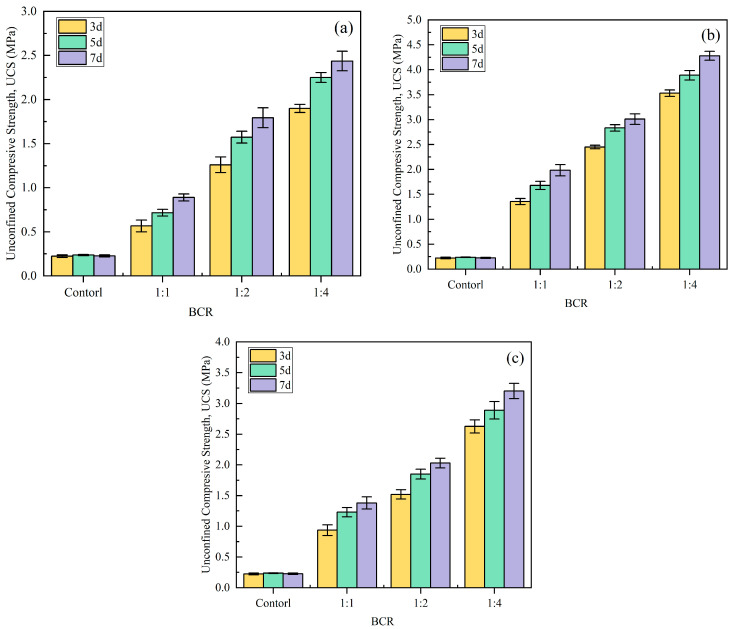
Influence of different factors on the UCS of bio-cemented sand: (**a**) *w* = 8%; (**b**) *w* = 12%; (**c**) *w* = 16%.

The unconfined compressive strength (UCS) serves as a widely recognized standard metric for quantifying the mechanical integrity of bio-cemented soils [52]. The variation in UCS of bio-cemented sand columns under different curing conditions is shown in Figure 8. It can be seen that the UCS of bio-cemented soils is always higher than that of the uncemented sand (0.24 MPa). The ratio of BCS played a significant effect on the UCS of bio-cemented sand columns, which increased with the increase in the proportion of CS. This may be due to the high UCS value of sand samples treated with a high CS concentration [39]. Some studies have shown that excessive concentration of calcium ions in the CS may inhibit the activity of urease to a certain extent, which may affect the curing effect. Therefore, from the perspective of reinforcement effect and economy, the concentration of CS should not be too high. In addition, UCS increases with the extension of the curing time. The UCS at the 5-day and 7-day curing age was improved by approximately 10.02–31.32% and 21.25–57.06%, respectively, compared to the 3-day age. However, there was not always a positive correlation between the UCS and bio-cured solution content, and the UCS showed an increasing and then decreasing trend when the range of bio-cured solution content was 8–16%, which was consistent with the findings in [53,54]. This suggests that there is a close relationship between the UCS and the degree of saturation, and that the formation of CaCO_3_ at the contact points between particles was more intensive under lower saturation conditions, and excessive levels of bio-cured solution can lead to a reduction in soil strength. The optimal water content of the aeolian sand used in this test is around 12%, and the sand is easier to be compacted under this water content to achieve a larger degree of compactness. While when the water content is higher, the sand can easily be liquefied under the same compaction work, the sand is in a highly saturated state, and the internal pores are filled with the liquid, which leads to the contact between particles not being enough. As a result, the UCS is reduced for a total amount of bio-cured content of 16% compared with 12%. Overall, the UCS of bio-cemented sand demonstrates a significant improvement compared to uncemented sand. This is due to the fact that a certain amount of CaCO_3_ crystals were generated between the sand particles during the MICP process, which on the one hand act as a filler material to fill up the pore between the sand particles, and on the other hand, as a binding material to bind and associate the sand particles, leading to a stronger structure and integrity in sand.

In this study, under the MICP formulation (12% bio-cured solution content and a BS-to-CS ratio of 1:4), the 7-day UCS of the bio-cemented sand reached 4.28 MPa. This value is approximately 10 times higher than the strength (450 kPa) of traditional cement-stabilized aeolian sand with 5% cement content reported in [55], and 4 times greater than the 7-day strength of aeolian sand stabilized under the optimal combination of fiber, silt, and cement (5% cement content, 12 mm fiber length, 2% fiber content, and 15% silt content) in the same study. Furthermore, it significantly exceeds the 7-day UCS (2.8 MPa) of cement–fly ash-stabilized aeolian sand gravel base courses with 66% aeolian sand content, as documented in [56].

### 3.2. Calcium Carbonate Content (CCC)

**Figure 9:**
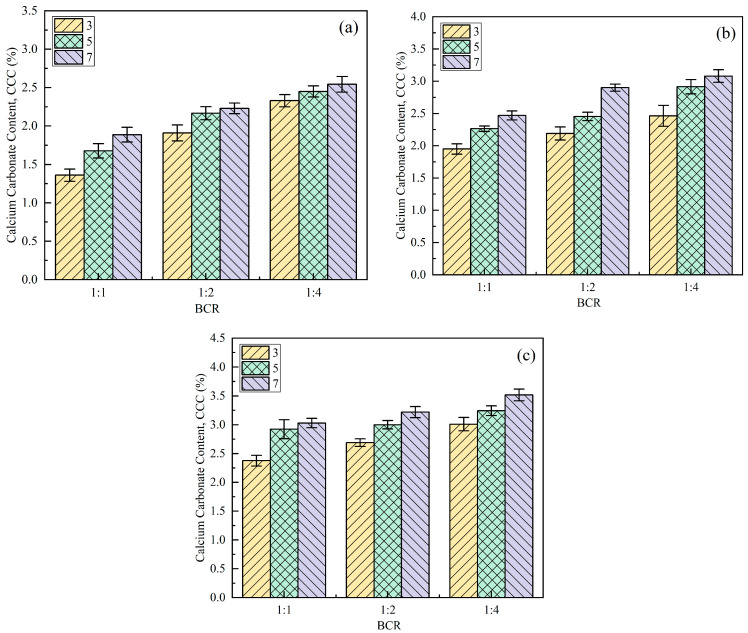
Influence of different factors on the *CCC* of bio-cemented sand: (**a**) *w* = 8%; (**b**) *w* = 12%; (**c**) w = 16%.

The formation of CaCO_3_ is the main reason for the improvement of bio-cemented soil properties [39]. Combined with Figure 9, it can be found that the change trend of *CCC* with the content of bio-cured solution and curing age is consistent with that of the UCS. When the content of the bio-cured solution ranged from 8% to 16%, the UCS showed a trend of first increasing and then decreasing, while *CCC* still increased with the increase in the total amount of bio-cured solution content, reaching a maximum of 3.52% at 16%. Based on the results of the UCS test and *CCC* determination, the optimal scheme of bio-cemented sand was determined, that is, the total amount of bio-cured solution content was 12%, the BCR was 1:4, and the curing age was 7 days; the UCS under this condition was 4.28 MPa, and the strength requirement of the cement stabilized material at 7 days was used as the base layer of highways and first-class highways under medium and light traffic (3–5 MPa) (JTG/T F20-2015) [57]. Therefore, this kind of sand can meet the design strength requirements of the road base in the desert area. Subsequent dynamic triaxial tests and road performance evaluations were conducted based on this stabilization scheme.

### 3.3. Compressive Failure Pattern

**Figure 10:**
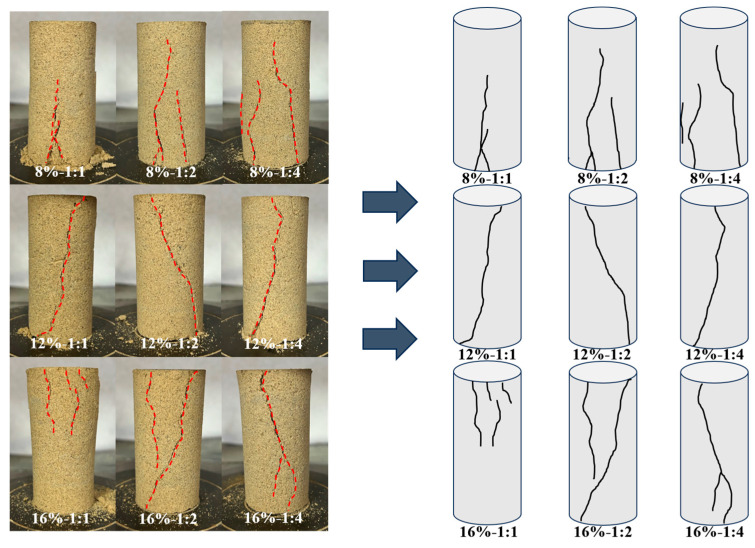
Damage patterns of aeolian sand samples under different curing conditions.

Typical failures of sand columns in the UCS tests under different curing conditions at the 7-day curing age are demonstrated in Figure 10. It can be seen that when the content of bio-cured content is 8%, the damage patterns of the samples are not significant, which mainly occurs at the bottom of the sand sample, and the failure pattern is conical damage. The damage degree of the sample is more intense at 12%, and the damage pattern is dominated by overall shear damage. While when the bio-cured content is 16%, the damage occurs mainly at the top of the sample, and the failure pattern is localized shear damage from the top. This indicates that when the bio-cured content is 12%, the sand column has the highest degree of consolidation and the integrity of the sample is increased. In addition, at any bio-cured content, the rupture surface of the samples gradually increased with the increasing percentage of CS, and the degree of damage was more drastic.

### 3.4. Dynamic Characteristics

#### 3.4.1. Cumulative Plastic Axial Strain (ε_p_)

**Figure 11:**
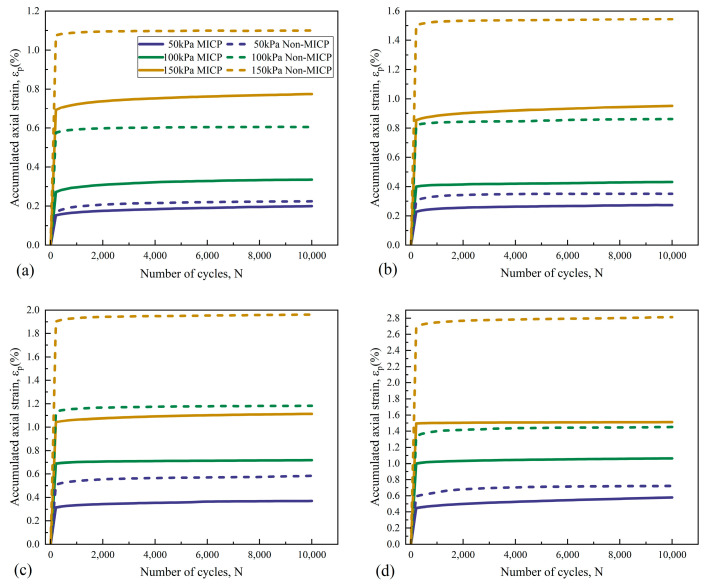
Relationship curves of *ε_p_-N* under different *CSRs*: (**a**) *CSR* = 1; (**b**) *CSR* = 3; (**c**) *CSR* = 5; (**d**) *CSR* = 7.

The axial deformation of the sand columns under cyclic loads consists of a recoverable elastic deformation and irrecoverable cumulative plastic deformation, in which the latter will have an unfavorable effect on the project when its value is too large [42]. The *ε_p_* is typically influenced by several factors such as the dynamic stress level and soil compactness [54,58]. The relationship curves of *ε_p_* with *N* for both bio-cemented and uncemented sand columns under cyclic loads are given in Figure 11. Due to the high overall strength of the dried sand column, the damage criterion of *ε_p_* = 5% was not reached after the test, but the pattern of the test results is obvious. Under the same dynamic stress level, the *ε_p_* of bio-cemented and uncemented sand columns has a consistent trend with the increase in *N*, which was that *ε_p_* grew rapidly with the increase in *N* first, and the particles inside the sand gradually became compact; then, the growth rate slowed down, and finally tended to stabilize after reaching a certain amount of *N*. This kind of relationship curve of *ε_p_* with *N* was shown to be a stabilized type of relationship curve. Both the *σ*_3_ and the *CSR* have a certain degree of influence on the *ε_p_* of the AS, and the *ε_p_* increases significantly with the increase in *σ_3_* for the same *N* and *CSR*. Meanwhile, the *ε_p_* of the bio-cemented sand columns also increases with the increase in *CSR*, which is the same trend as that of the uncemented sand columns. Under the same loading condition, the *ε_p_* of the cemented sand column remains consistently lower than that of the uncemented sand column, which indicates that the MICP cementation effectively improves the strength of the soil and reduces the *ε_p_* of the aeolian sand under cyclic loading. Furthermore, under identical loading conditions (*N* = 10,000, *σ_3_*= 50 kPa, *CSR* = 5), the bio-cemented sand exhibited a plastic strain (*εₚ*) of approximately 0.37%. This value is lower than the *εₚ* of 0.5% reported for the 20% *FC*–8% *GP* stabilized aeolian sand specimen by Chen et al. [42], and is also lower than the value of 0.52% observed in 4% cement-stabilized aeolian sand after 2000 loading cycles [48].

#### 3.4.2. Dynamic Elastic Modulus (E_d_)

Soil stiffness is used to measure the ability of the material to resist elastic deformation, and the change law of soil stiffness under cyclic loads is usually reflected by the parameter of *E_d_* [49], which represents the relationship between axial dynamic stress (*σ_d_*) and axial dynamic strain (*ε_d_*); it can be calculated by Equation (5):

(5) $$E_{d}=\frac{\sigma_{d}}{\varepsilon_{d}}\times 100\%$$

**Figure 12:**
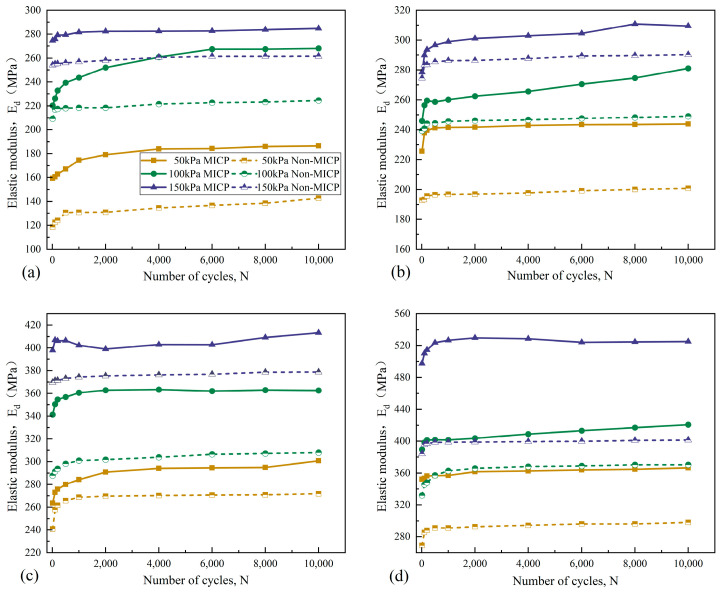
Relationship curves of *E_d_-N* under different *CSRs*: (**a**) *CSR* = 1; (**b**) *CSR* = 3; (**c**) *CSR* = 5; (**d**) *CSR* = 7.

The relationship curves of *E_d_* with *N* for both bio-cemented and uncemented sand columns under cyclic loads are given in Figure 12. The change trends in *E_d_* with *N* for the two sand columns were also close to the same; specifically, *E_d_* increases slowly with the increase in *N* first, which is related to the large amount of accumulated axial plastic strain generated in the early stage of the sand columns, and when a certain value of *N* is reached, the *E_d_* gradually tends to be stabilized, ultimately remaining basically unchanged until the end of the test. This is due to the fact that with the increase in *N*, the sand particles are gradually compacted and its density increases, which increases the occlusal force and the friction force between the sand particles; thus, the ability to resist deformation is also enhanced. The *E_d_* increases with the increase in *σ_3_* under the same *CSR*. This is because when the *σ_3_* is small, the strain of sand is mainly manifested as a rapidly developing plastic strain, and with the increase in *σ_3_*, due to its “hoop” effect [59], the lateral restraint of the sand columns increases. Therefore, the sand particles become more compact due to extrusion, and the contact area between sand particles increases, which gives the sand better integral properties, thereby slowing down the development of the plastic strain, and then improving the *E_d_* of bio-cemented sand samples. The *E_d_* increases with the increase in the *CSR* under the same *σ_3_*. This is because the larger the value of the *CSR* is, the larger is the vertical load acting on the sand column, and the densification of the sand column gradually increases; as a result, the stiffness increases under cyclic loads.

The improvement effect of the cementation of MICP on the resistance to deformation is always present under any loading condition. This may be due to the fact that, on the one hand, the MICP treatment improves the internal structure of the AS, enhances the cementation connection between the particles, reduces the development of cumulative deformation, and also has a certain inhibition effect on the elastic deformation. On the other hand, compared with the cumulative deformation, the elastic deformation of the soil under cyclic loading is relatively small and recoverable, so that the ability of the bio-cemented sand to resist the cumulative deformation appears to be more prominent. The *ε_p_* and *E_d_* of the sand column have good positive correlation with both *σ_3_* and *CSR* levels. Under the conditions of *σ_3_* = 150 kPa and *CSR* = 7, the *ε_p_* and *E_d_* of the bio-cemented sand reached the maximum values (*ε_p_* = 1.51%, *E_d_* = 525.18 MPa), respectively, and the *ε_p_* of the bio-cemented sand decreased by 46% while the *E_d_* increased by 31% approximately compared with uncemented sand.

### 3.5. Road Performance Indicators

#### 3.5.1. Splitting Tensile Strength

**Figure 13:**
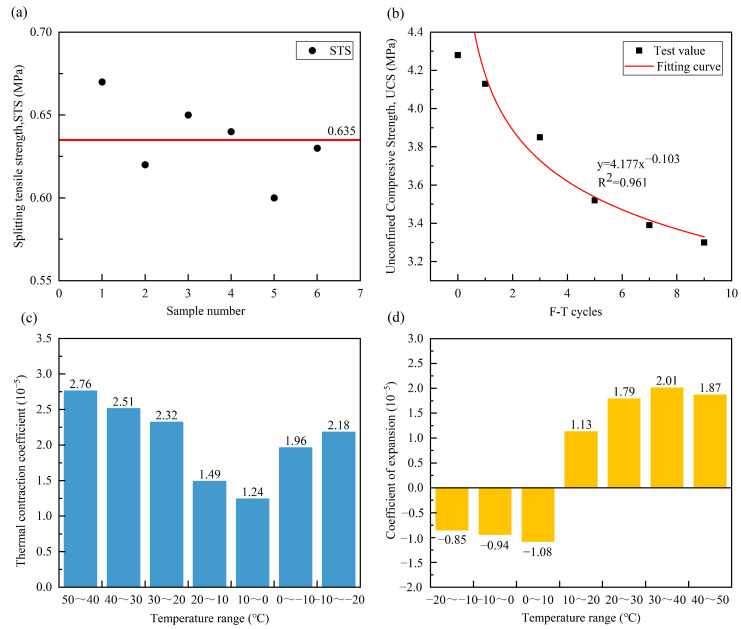
Road performance index of bio-cemented sand: (**a**): Splitting tensile strength; (**b**): Frost resistance; (**c**): Temperature shrinkage coefficient; (**d**): Coefficient of expansion.

The splitting tensile strength has been commonly used to characterize the indirect tensile properties of inorganic bonded stabilized base layers under the combined action of tension and compression. Given the current absence of standardized specifications governing bio-cemented sand applications in pavement engineering, this study takes the specifications and research results related to inorganic binder stabilization materials, which are the most widely used in engineering, as a reference to make a preliminary evaluation of the splitting tensile strength value of bio-cemented sand columns. Figure 13a illustrates the strength of bio-cemented sand under its optimal curing condition. The bio-cemented sand column exhibits an average strength of 0.635 MPa, which exceeds the value (0.61 MPa) of cement fly ash stabilized AS gravel subgrade (66% AS content, 7-day curing ages) in [56], and the strength (0.63 MPa) of CTSF-19 fiber reinforced cement reinforced sand subgrade (28-day curing ages) in [60], which is a medium-high strength level in its type of study. This indicates that the MICP treatment improves the cohesion of the AS, which causes the bio-cemented sand to have better tensile properties, and the value of strength achieved in this test even exceeds the design strength requirements of cement stabilized material (0.4–0.6 MPa) [61], which can meet the splitting tensile strength requirements of the road base.

#### 3.5.2. Frost Resistance

Due to the perennial freeze–thaw cycles experienced by AS in desert regions with large diurnal temperature variations, studying the frost resistance of bio-cemented sand is essential. Figure 13b illustrates the UCS of bio-cemented sand under different freeze–thaw cycles. Data fitting reveals an exponential relationship between strength and freeze–thaw cycles, where the strength declines rapidly initially and then gradually stabilizes with increasing cycles. This phenomenon is attributed to continuous freeze–thaw cycling causing tensile stresses in the cemented positions of CaCO_3_ crystals on the sand bioparticles, leading to microcracks in the bio-cemented sand column, thereby weakening the soil strength [62,63]. After nine freeze–thaw cycles, the samples exhibited a strength loss rate of 22.9%, while still maintaining a compressive strength of 3.3 MPa, demonstrating the material’s favorable frost resistance.

#### 3.5.3. Temperature Shrinkage and Expansion Coefficient

The deformation of the road base caused by factors such as high and low temperature cycles in desert areas has become one of the common diseases in road engineering. Therefore, by carrying out the temperature shrinkage and arch expansion tests under extreme temperature gradient conditions, the temperature shrinkage coefficient and expansion coefficient of bio-cemented sand are obtained as shown in Figure 13c,d, respectively. It can be seen from Figure 13c that with the gradual decrease in the ambient temperature, the temperature shrinkage coefficient of bio-cemented sand shows a trend of first decreasing and then increasing, and the temperature shrinkage coefficient reaches its lowest when the temperature range is 10 °C~0 °C, which indicates that the sample is in a state of continuous shrinkage during the cooling process, and in the range of 10 °C~0 °C, due to the coupling of various factors such as the change in the substrate shrinkage moisture state and the change in the morphology of the cemented substance, the temperature shrinkage coefficient of this interval is the smallest. It may be a temperature zone where the material behaves relatively steadily and can be used as a reference window for construction or maintenance. From Figure 13d, with the gradual increase in ambient temperature, the expansion coefficient of bio-cemented sand decreases first and then increases, among which, when the temperature range is −20 °C~10 °C, the expansion coefficient of the solidified sand is negative, and when the temperature range is 10 °C~50 °C, the expansion coefficient is positive. This shows that the sample shows a tendency to shrink first and then expand during heating. Combined with the temperature contraction test and arch expansion test, it can be seen that the rise and fall in the ambient temperature has a greater impact on the deformation of the bio-cemented sand, and attention should be paid to the thermal insulation of the grass-roots level in the later road construction and operation process, or can be combined with other low-temperature-sensitive materials for joint curing treatment of AS. The bio-cemented sand demonstrated significantly superior pavement performance compared to traditional cement-based stabilization materials [64,65].

### 3.6. Microstructural Characteristics

**Figure 14:**
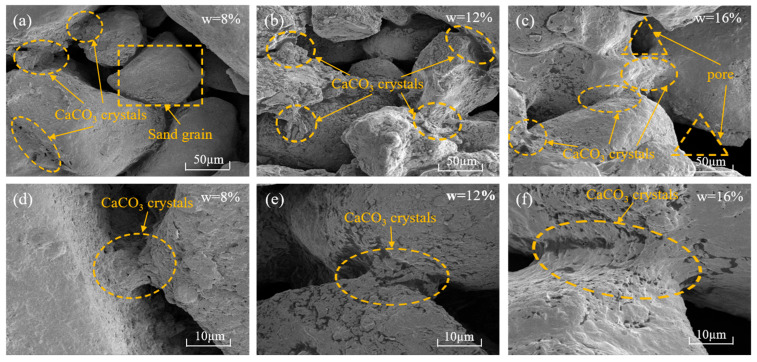
Scanning electron microscope (SEM) image of MICP bio-cemented sand: (**a**–**c**): *w* = 8%, 12%, 16% (1.00 kx); (**d**–**f**): *w* = 8%, 12%, 16% (5.00 kx).

The SEM images of the bio-cemented sand under varying bio-cured solution contents (*w* = 8%, 12%, 16%), 1:4 volume ratio of BS to CS and 7-d curing age were taken at the magnification of 1.00 kx and 5.00 kx, as shown in Figure 14. It can be seen that with the increase in the bio-cured solution content, the amount of CaCO_3_ crystals formed between the sand particles increases significantly, the size of CaCO_3_ crystals increases, and the cementation attachment becomes more and more tight. A small number of scattered and irregularly shaped CaCO_3_ crystals embedded between the bio-cemented sand particles with 8% bio-cured solution content can be seen in Figure 14a, and the intergranular connections are relatively loose. Figure 14b demonstrates that CaCO_3_ crystals form an orderly layered structure within the interparticle pores of sand particles, resulting in a robust interlocking configuration. However, as shown in Figure 14c, the bio-cemented sand column with 16% bio-cured solution content has more pores inside the sand column by the evaporation of water during the later drying process, which makes its integral strength decrease compared with the bio-cemented sand column with 12% bio-cured solution content. As can be clearly seen from Figure 14d–f, the CaCO_3_ crystals generated by the MICP process act as a “biological bridge” to connect the sand particles [66], which significantly enhances both the compressive strength and structural stiffness of the bio-cemented sand columns.

## 4. Discussions

Based on the comparative analysis of solidification methods in this study and similar research (Table 4), microbial-induced calcium carbonate precipitation (MICP) technology demonstrates significant advantages over other mainstream techniques for stabilizing aeolian sand. The MICP method primarily relies on biocementation to produce solidified sand with high strength and durability comparable to traditional cement-based materials. Furthermore, it exhibits notable environmental friendliness, largely because the process occurs at ambient temperatures and avoids the high carbon emissions associated with cement production [2]. This positions MICP as a more sustainable alternative for geotechnical applications where environmental impact is a critical consideration. However, high costs and potential environmental pollution from ammonia and nitrogen byproducts remain key challenges hindering its large-scale commercialization. In contrast, colloidal silica [67] offers environmental benefits and excellent permeability but suffers from limited cementation strength. Similarly, geopolymers [42] leverage waste utilization, low hydration heat, and low shrinkage yet face issues related to the corrosiveness of alkaline activators and high expenses. Methods using fibers [68] or biopolymers [69] excel in operational simplicity and environmental compatibility but are fundamentally constrained by modest improvements in unconfined compressive strength and unresolved long-term durability concerns under humid conditions.

Analysis confirms that while no single technology is universally superior, MICP achieves a powerful combination of high strength and low carbon footprint that other methods cannot simultaneously deliver. Therefore, this study is essential as it systematically evaluates the feasibility of using bio-cemented sand as a road base material in terms of mechanical strength, dynamic durability, and road performance, and it provides a clear basis for ongoing research. Future efforts should focus on addressing the issues of cost and ammonia production associated with MICP to facilitate its broader application in large-scale geotechnical engineering projects.

## 5. Conclusions

In this study, an attempt was made to use bio-cemented sand as a road base material and the feasibility of this approach was explored by conducting various laboratory mechanical tests. The specific conclusions of this study are as follows:

(1) This study demonstrates the significant potential of bio-cemented sand as a sustainable and effective material for road base construction. The key finding is that microbially induced calcite precipitation (MICP) treatment can enhance the mechanical properties and durability of aeolian sand to meet or exceed standard road base requirements. Specifically, an optimal MICP treatment condition was identified (12% bio-cured solution content, BS:CS ratio of 1:4, and 7-day curing age), under which the bio-cemented sand achieved a maximum UCS of 4.28 MPa, along with improved resistance to cyclic loading.

(2) Beyond the mechanical performance demonstrated in laboratory tests, the material exhibited favorable frost resistance, making it suitable for construction in cold regions. However, it shows moderate thermal sensitivity under large temperature variations, and it is recommended to incorporate low-thermal-sensitivity materials such as nanomaterials and clays for co-modification treatment during construction. Microscopic analysis further confirmed that the strength improvement is primarily attributed to the pore-filling effect of calcium carbonate precipitation, while also revealing that an excessively high solution content leads to pore formation and strength reduction during the drying process.

(3) Despite these promising results, this study has several limitations. The tests were conducted under controlled laboratory conditions, which may not fully represent complex field environments. Moreover, the long-term durability and performance of bio-cemented sand under real-world traffic and environmental conditions remain unverified. The economic feasibility and scalability of the MICP treatment process for large-scale construction projects also require further assessment.

(4) Future research should focus on field trials to validate laboratory findings and assess in situ performance. Investigations into the long-term behavior of bio-cemented sand under continuous traffic loading and environmental exposure are essential. Additionally, efforts to optimize the treatment process for cost efficiency and large-scale application would be valuable to promote practical implementation.

## Figures and Tables

**Table 1:** Physical property indexes.

Maximum Dry Density (g·cm^−3^)	Optimum Water Content (%)	Specific Gravity	Coefficient of Uniformity	Coefficient of Curvature
1.91	12	2.60	2.75	0.92

**Table 2:** Types of MICP bio-cemented sand columns.

Bio-Cured Solution Content (%)	BCR	Curing Age (d)
8, 12, 16	1:1, 1:2, 1:4	3, 5, 7

BCR = the ratio of bacterial solution (BS) to cementation solution (CS).

**Table 3:** Dynamic triaxial test details.

Types of Samples	*σ_3_* (kPa)	$q^{ampl}$ (kPa)	*CSR*	ƒ (Hz)	*N*
MICP/Non-MICP	50	50	1	1	10,000
	100	300	3	1	10,000
	150	750	5	1	10,000
	50	350	7	1	10,000
	100	100	1	1	10,000
	150	450	3	1	10,000
	50	250	5	1	10,000
	100	700	7	1	10,000
	150	150	1	1	10,000
	50	150	3	1	10,000
	100	500	5	1	10,000
	150	1050	7	1	10,000

**Table 4:** Summary of advantages and disadvantages of the stabilized sand in this study compared to similar studies.

Sand Type	Stabilization Materials or Techniques	Interaction with Soils	Advantages	Disadvantages	References
Aeolian sand	MICP	CaCO_3_ cementation and filling	High strength, eco-friendly, good durability	High cost, by-product ammonia nitrogen may cause environmental pollution	This study
F161 sand	Colloidal nano-silica hydrosols	Gel cementation and filling	Good liquefaction resistance, eco-friendly	Limited cementation strength, high cost, long-term durability requires further validation	[67]
Aeolian sand	Cement	Cement hydration reaction	Technologically mature, high strength	Brittle failure prone to shrinkage cracking, high carbon emissions	[2]
Aeolian sand	Geopolymers	Geopolymerization reaction	Waste utilization, low heat of hydration, low shrinkage	High cost, the strong alkaline activator is corrosive	[42]
River sand	Fibers	Physical encapsulation and restraint	High toughness and tensile strength, simple construction process, eco-friendly	Low unconfined compressive strength, difficulty in ensuring uniform fiber distribution, long-term durability requires further validation	[68]
Silty Sand	Biopolymer	Physical encapsulation and hydrogen bonding	Improved eco-friendly, water retention capacity, simple application process	Poor water resistance and long-term durability, limited strength	[69]
Aeolian sand	Chemical emulsions	Physical adsorption and adhesion	Good water resistance and flexibility	Limited strength, high construction sensitivity, environmental pollution	[7]

## Data Availability

The original contributions presented in this study are included in the article. Further inquiries can be directed to the corresponding author.
